# Punishment: one tool, many uses

**DOI:** 10.1017/ehs.2019.12

**Published:** 2019-11-12

**Authors:** Nichola J. Raihani, Redouan Bshary

**Affiliations:** 1 Department of Experimental Psychology, University College London, 26 Bedford Way, London WC1H 0AP, UK; 2 Institut de Biologie, Université de Neuchâtel, Rue Emilie-Argand 11, Neuchâtel, CH-2000, Switzerland

**Keywords:** Competition, cooperation, fairness, punishment, spite

## Abstract

Humans are outstanding in their ability to cooperate with unrelated individuals, and punishment – paying a cost to harm others – is thought to be a key supporting mechanism. According to this view, cooperators punish defectors, who respond by behaving more cooperatively in future interactions. However, a synthesis of the evidence from laboratory and real-world settings casts serious doubts on the assumption that the sole function of punishment is to convert cheating individuals into cooperators. Instead, punishment often prompts retaliation and punishment decisions frequently stem from competitive, rather than deterrent motives. Punishment decisions often reflect the desire to equalise or elevate payoffs relative to targets, rather than the desire to enact revenge for harm received or to deter cheats from reoffending in future. We therefore suggest that punishment also serves a competitive function, where what looks like spiteful behaviour actually allows punishers to equalise or elevate their own payoffs and/or status relative to targets independently of any change in the target's behaviour. Institutions that reduce or remove the possibility that punishers are motivated by relative payoff or status concerns might offer a way to harness these competitive motives and render punishment more effective at restoring cooperation.

## Graphical abstract

**Figure:**
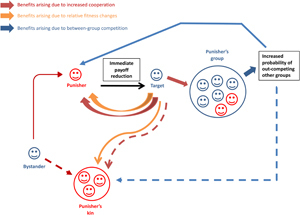

**Media summary:** Punishment is thought to be a powerful driver of human cooperation but it does not often convert defectors into cooperators. In addition, punishment is frequently aimed at cooperators, or individuals who did nothing wrong, and often prompts retaliation rather than cooperation. We highlight what we believe is a relatively neglected role of punishment: it is a form of competition, whereby punishers equalise or elevate their own payoffs in relation to others. Cultural institutions that limit potential for competitive punishment might have played a vital role in harnessing humans’ competitive motives and rendering punishment more effective at restoring cooperation.

## 1. Introduction

In what has become a classic study, Clutton-Brock and Parker (1995) defined punishment as occurring when: (a) one individual cheats by performing an action that lowers the partner's payoff, relative to alternative actions; (b) the harmed individual incurs a temporary payoff reduction to reduce the payoffs of the cheating individual; (c) the target then behaves more cooperatively in future interactions with the punisher and hence causes the punisher's payoffs to increase. This definition overlaps with what Boyd and Richerson (1992) earlier called ‘retribution’. Common to both definitions is the idea that there is (a) a cause, (b) the punishment act and (c) the future benefits of the act that explain why punishment is under positive selection. We note that some authors have argued that punishment can also include acts that do not require the punisher to experience any immediate costs (Boyd and Richerson 1992; Nakao and Machery 2012), as is the case with withholding benefits from, or reciprocally exploiting, cheats (Bhui *et al*. 2019; Boyd 2017). While we are sympathetic to the fact that the colloquial use of the word punishment may admit such cases, these scenarios are relatively unproblematic to explain from an evolutionary perspective. More puzzling is when and why a tendency to invest in behaviour directed at harming others can ever be under positive selection – and as such we prefer to stick with definitions that assume that punishment is at least temporarily costly to administer (Úbeda and Duéñez-Guzmán 2011).

Although punishment is undoubtedly more common among humans than non-human species (Raihani *et al*. 2012a), it has been documented in a handful of species, such as among cleaner fish and their reef-fish clients, among male–female pairs of cleaner fish and in vervet monkeys (Arseneau-Robar *et al*. 2016, 2018; Bshary and Grutter 2002; Raihani *et al*. 2010). In the cleaner fish mutualism, clients may use punishment to make cleaners eat their ectoparasites rather than their protective mucus, which is preferred by cleaners (Grutter and Bshary 2003). A second form of punishment is administered by male cleaners towards their female partners if the latter cheat a shared client (Raihani *et al*. 2010, 2012b). Finally, in vervet monkeys, males and females of the same group may punish each other during intergroup encounters (Arseneau-Robar *et al*. 2016), with males punishing females who initiate attacks (Arseneau-Robar *et al*. 2018) and females punishing males who do not participate. While the focus in this paper is on punishment in humans, it will be helpful to keep these examples in mind, as we shall return to the ways that punishment in humans might (or might not) differ from what we see in other species in our concluding remarks.

Some of the first work to experimentally explore punishment in humans was done in laboratory studies using economic games (Fehr and Gächter 2000, 2002a; Yamagishi 1986). These classic studies showed that people will pay for a sanctioning mechanism (Yamagishi 1986) or will take it upon themselves to punish co-players who do not contribute to a public good (Fehr and Gächter 2000, 2002a), even though the costs of punishment are personalised and the benefits of punishment (in terms of increased contributions to the public good) are shared among punishers and non-punishers alike. The findings of these studies lent credence to theoretical arguments that punishment can (sometimes) be viewed as a second-order public good (Boyd *et al*. 2003; Boyd and Richerson 1992), although subsequent work suggested that punishment might more often fit the payoffs of the volunteer's dilemma (Raihani and Bshary 2011), where investments can be self-serving rather than altruistic. Nevertheless, these early studies have been highly influential in informing theories of human cooperation. Nevertheless, the conclusions have since been challenged in two ways. First studies on non-WEIRD subjects (where WEIRD stands for Western, Educated, Industrialised, Rich, and Democratic; Henrich *et al*. 2010a, b; Jones 2010) and studies conducted in field settings suggest that people may not be as willing to punish as the laboratory studies with Western undergraduates imply. Second, many follow-up laboratory studies on WEIRD subjects that alter important details of the experimental design and that study the motives underlying punishment in more detail have produced diverging results. These discrepancies are potentially important since many accounts of the evolution of cooperation in humans have invoked punishment as a key supporting mechanism (Boyd *et al.* 2003; Chudek and Henrich 2011; Gintis *et al.* 2003; Raihani *et al.* 2012a; Raihani and Bshary 2015a).

We start by reviewing the literature on the behavioural and economic consequences of punishment in economic games, as well as a selective survey of data from punishment in the real world. Our aim is not to provide a comprehensive review of ethnographic observations but rather to provide an illustrative overview of key patterns and to identify open questions in the field. We will outline an alternative functional hypothesis for punishment, which we call the ‘competitive function’, arguing that this has been relatively understudied and yet deserves more theoretical and empirical attention. Finally, we discuss the different empirical predictions that can be made based on different functional accounts of punishment.

## 2. The evolution of punishment

In humans, punishment has often been studied in the context of stylised laboratory games (Chaudhuri 2011; Guala 2012), where individuals can pay a small monetary cost to inflict a (usually) larger fine on a target. These laboratory games have emphasised people's willingness to punish others for transgressions (Fehr and Gächter 2000, 2002a; Yamagishi 1986), including when they were not the victim (‘third-party punishment’; Fehr and Fischbacher 2004). In the case of second-party punishment, where the punisher was the victim (or one victim) of the cheating individual, it is often assumed that the punisher can reap a return on investment because of a change in the target's behaviour (e.g. Clutton-Brock and Parker 1995), or because a bystander observes the punishment and this deters the bystander from defecting when they interact with the punisher (dos Santos *et al*. 2013). Third-party punishment is thought to be more puzzling from an evolutionary perspective, but this is largely due to the assumption that the third party will not interact with the target of punishment again in the future. This assumption – like second-party punishment in one-round games – makes it more difficult to reconcile the costly act of punishment with the possibility for downstream benefits for the punisher. Recent theoretical work has indicated that third-party punishers may nevertheless benefit when their punitive reputation is known to future interaction partners, which is likely when populations are relatively tightly structured and viscous (Roos *et al.* 2014). Under these circumstances, the possibility for future interactions with the target of punishment or with bystanders who observe the punishment act seems to be a more important factor in understanding how punishers can be compensated for their investments, than whether the punisher was the original victim of the cheating individual or not.

Before discussing the empirical literature, it is worth bearing in mind that many of the studies we cite were conducted in the laboratory using WEIRD participants (but see for notable exceptions Ensminger and Henrich 2014; Gächter and Herrmann 2011; Henrich 2000; Henrich *et al*. 2010c, 2005, 2006; Herrmann *et al*. 2008; Marlowe 2009; Marlowe *et al*. 2008; Marlowe, *et al*. 2011; Wiessner 2009). In contrast to the laboratory studies, data from the real world suggest that people are often unwilling to punish when opportunities to do so arise (Balafoutas *et al.* 2014a; Baumard 2010; Guala 2012; Pedersen *et al*. 2018; but see Mathew and Boyd 2011). The relatively high levels of punishment observed in laboratory games might therefore be an artefact that stems from the paucity of alternative options offered to participants (e.g. see Raihani and Bshary 2015b). In real-world settings, where alternative options are available (and where people might be involved in more than one kind of social interaction with the same partner), people can withhold opportunities to help rather than actively harm transgressors (Balafoutas *et al*. 2014a), can ‘vote with their feet’ by either expelling or distancing themselves from offenders or free-riders (Boehm 2001; Lee 1979) or can use restorative justice that focuses on compensating victims for harm incurred rather than imposing penalties on the wrongdoers themselves (Hirsch *et al*. 2003; Pupu and Wiessner 2018; Wiessner and Pupu 2012). In addition, work that has been conducted in non-industrial, small-scale societies typically finds that people living in such societies are less willing to invest in punishment of others even in the context of artificial laboratory games (Henrich *et al*. 2010a; Wiessner 2009) and that investment in third-party punishment in particular is less common (Marlowe *et al*. 2011). These caveats should be borne in mind when assessing the evidence below.

**Figure 1.**
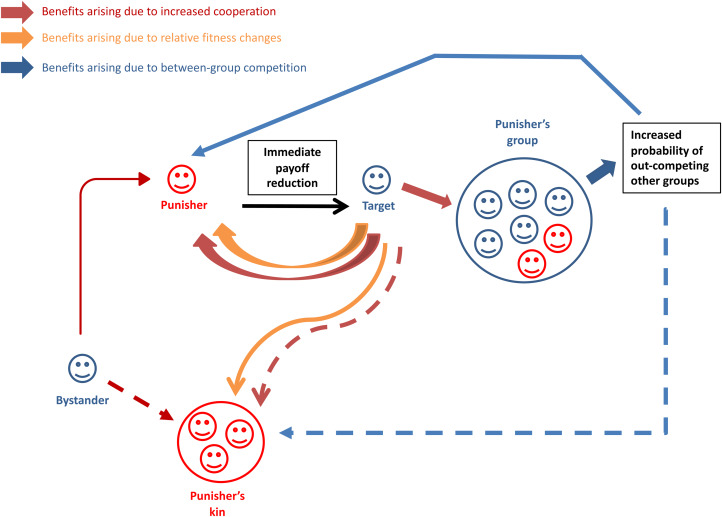
Possible feedback loops by which punishment could yield inclusive fitness benefits to punishers. Red arrows denote benefits that arise because punishment causes the target or bystanders to cooperate more (with dashed lines indicating that the cooperation is directed at punisher's kin). Orange arrows denote benefits that arise because punishment changes the relative payoff difference between punisher and target. This feedback loop assumes that competition is relatively local. Blue arrows denote benefits that arise because competition between groups is stronger than competition within them. Blue arrows therefore assume that competition is relatively global.

Although theoretical work has emphasised that punishment can stabilise any behaviour within a group (Boyd and Richerson 1992), researchers interested in the evolution of cooperation have been especially interested in the idea that punishment can act as a tool to promote cooperation (the pathway denoted by the red arrows in Fig. 1). Under the assumption that punishment involves paying a cost to harm another individual, the higher fitness payoffs that result from the target's increased cooperation offers one plausible mechanism by which such investments can be favoured by selection (Boyd and Richerson 1992; Clutton-Brock and Parker 1995; Raihani *et al*. 2010). Punishers might additionally benefit because their actions are witnessed by other individuals who then behave more cooperatively with the punisher in future (dos Santos *et al*. 2011; dos Santos and Wedekind 2015; Jordan and Rand 2017; Raihani and Bshary 2015b, c). Nevertheless, in laboratory games, humans willingly invest in punishment in scenarios where the act is not observed by others, where the punisher will not interact with the target again in future, and/or where the benefits will be shared by other individuals, including non-punishers. In such cases, punitive preferences could reflect a mismatch between the laboratory environment and a human psychology that evolved in environments where interactions are truly one-shot and/or unobserved (e.g. Cosmides and Tooby 1997; Tooby *et al*. 2006, but see Fehr and Henrich 2003; Hill *et al*. 2014; Raihani and Bshary 2015a) or could instead reflect a psychology that has been under positive selection because of the effect punishment has on group success in the context of between-group competition (e.g. Boyd *et al*. 2003; blue arrows in Fig. 1).

Here, we want to highlight what we believe is a neglected strategic goal of punishment: to compete with others, rather than to encourage future cooperation. This possibility was mentioned as a footnote in Fehr and Gächter (2000) (see also Baumard 2010; Boehm 2001; Cosmides *et al*. 2002; Nakao and Machery 2012; Saaksvuori *et al*. 2011). It also has similarities to the theory of inequity aversion proposed by Fehr and Schmidt (1999) as well as the levelling-down strategies used in traditional societies, discussed in Boehm (2001) in that, like the competitive punishment hypothesis, both of these theories emphasise that people should be sensitive to their own payoffs (or status) relative to others’. Previous theoretical models have shown that punishment strategies that respond to relative payoffs (so-called ‘egalitarian’ punishment strategies) can be equally or even more effective than punishment strategies that rely on observing or correctly inferring the target's strategy (Scheuring 2010; Tamura *et al*. 2011) and we agree with Scheuring (2010) that the assumption that punishers do always know the strategies of players they interact with is problematic when one considers that this may not always (or even often) be the case in the real world. Additionally, a recent theoretical model on the evolution of cooperation (which was modelled as respect for resource possession) found that cooperation was stabilised when resource holders (or other individuals) responded to ‘raiding’ (attempts to steal the resource) with retaliation (i.e. punishment). Nevertheless, when the level of inequality between resource-holders and resource-seekers exceeds a critical threshold (which can be thought of as akin to variation in payoff differences among players in a laboratory game), then cooperation collapses and raiding strategies come to dominate the population until this inequality is reduced. This model, although not explicitly framed in terms of cooperation and punishment, illustrates the evolutionary logic of considering relative payoff differences in understanding the evolution of cooperation and punishment in nature.

A payoff-levelling goal might also reflect an alternative evolutionary pathway for punishment: under local competition, it could be favoured simply because it allows punishers to reduce or remove fitness differentials between themselves and targets (Cosmides *et al*. 2002), to elevate their fitness above targets or to gain indirect fitness benefits if their kin are the main beneficiaries of the target's reduced competitiveness. The latter scenario, where the actor incurs lifetime fitness costs to impose fitness costs on another individual, fits the Hamiltonian definition of spite (Gardner and West 2004). In these scenarios (orange arrows in Fig. 1), no wrong-doing or altering of future behaviour of the target is necessary to explain the act of punishment and any behaviour change effect of punishment would be an exaptation. Importantly, the payoff consequences of acting upon punitive sentiments within the context of experimental games do not capture the direct and/or indirect fitness consequences of punishment: preferences for punishment could have been favoured by selection not because punishment tends to change targets’ behaviour but because it tends to leave the punisher (and/or his relatives) at a relative advantage. Under this view, punishment need not be restricted to those who were the victims of the original cheating individual, and could be favoured even when the punisher was an uninvolved third party.

In laboratory settings, this possibility for competition is often built into the design of the game since the cost of punishment to the punisher is usually smaller than the negative impact on the target. This confound is especially acute in studies that use variable fee-to-fine ratios (e.g. as in Denant-Boemont *et al.* 2007; Faillo *et al.* 2013; Fehr and Gächter 2000; Masclet *et al.* 2003; Nikiforakis 2008a), where the punishment reduces the target's earnings by a percentage rather than a fixed sum. Under variable fee-to-fine ratios, punishers can have a larger impact on the payoffs of defectors than on the payoffs of other cooperators (as pointed out by Casari 2005).

Below we outline the key predictions made by the hypothesis that punishment is (primarily) a tool to promote cooperation, along with a brief summary of the theoretical and empirical evidence that supports or undermines each prediction.

### Prediction 1: Punishers should also be cooperative individuals

In many models, the tendency to punish defectors is assumed to be linked to the tendency to cooperate, meaning that a punisher can also be assumed to be a cooperator (although the reverse is not necessarily true; Andrés Guzmán *et al.* 2007; Bowles and Gintis 2004; Boyd *et al*. 2003; Boyd and Richerson 1992; Henrich and Boyd 2001, but see Huang *et al*. 2018; Eriksson *et al.* 2014; Lehmann *et al*. 2007; Úbeda and Duéñez-Guzmán 2011). This assumption has also been described in terms of a psychological propensity to behave as a strong reciprocator (an individual that has a preference to conditionally cooperate and to punish non-cooperators; Gintis 2000). The theoretical basis of this assumption is that preferences for strong reciprocity reflect an underlying, more general preference for fairness. Individuals that have such fairness preferences are prepared to incur personal costs to prevent unequal outcomes from occurring or to reduce the extent of inequality (Fehr and Schmidt 1999). Nevertheless, several lines of empirical evidence refute this prediction. For example, in a one-shot public goods game, the tendency to cooperate was unrelated to punitive tendencies: free-riders punished just as much as cooperators in this setting (Weber *et al.* 2018). Similarly, using a series of social dilemma tasks, Yamagishi and colleagues (2012) found that the tendency to reject unfair offers in the Ultimatum Game (which has been interpreted as a form of costly punishment owing to strong reciprocity preferences; Fehr and Fischbacher 2003; Fehr and Gächter 2002b; Gintis *et al*. 2003) was unrelated to prosocial behaviour in other games (see also Albrecht *et al.* 2018; Brañas-Garza *et al.* 2014; Eriksson *et al.* 2014; Hoeft and Mill 2017; Kriss *et al.* 2016; Peysakhovich *et al.* 2014). Thus, the preference for cooperation and for punishment does not appear to be as tightly linked as theories based on strong reciprocity preferences imply, and evolutionary simulations on two-player interactions obtain as an outcome of natural selection (Wubs *et al*. 2016).

### Prediction 2: Punishment should cause targets to behave more cooperatively in future

In human studies, stylised economic games have been used to compare how cooperation levels differ as a function of whether punishment is possible or not. Evidence that cooperation is higher when punishment is possible (compared with when it is not) has often been taken as evidence that punishment causes cheaters to cooperate in future rounds (Ambrus and Greiner 2012; Cinyabuguma *et al.* 2006; Fehr and Gächter 2000, 2002a; Fischer *et al*. 2016; Masclet *et al.* 2003; Ones and Putterman 2007; Page *et al.* 2013; Pfattheicher *et al.* 2018; Reuben and Riedl 2009; Rockenbach and Milinski 2006; van Miltenburg *et al.* 2017). However, we suggest that this conclusion is premature because the evidence is correlational, with several alternative explanations that need to be excluded.

First, in some studies, analyses showing that targets who are punished in round *t* increase their investments in round *t* + 1 do not control for the mean level of cooperation in the group and thus do not rule out the possibility that participants are conditionally cooperating (by responding to the higher contributions of other group members) rather than responding to punishment (e.g. Fehr and Gächter 2000, 2002a; Pfattheicher *et al.* 2018; Rockenbach and Milinski 2006; Shinada and Yamagishi 2007). Studies in two-player settings indicate that the likelihood of a defector cooperating in round *t* + 1 hinges crucially on whether the partner cooperated in round *t* (Barclay and Raihani 2016; Bone *et al*. 2015, 2016). Once this conditional cooperation is accounted for, then the additional effect of punishment on inducing cheats to cooperate is absent (Barclay and Raihani 2016; Bone *et al*. 2015, 2016). Similar results have been obtained in the context of *n*-player games (de Melo and Piaggi 2015; Kirchkamp and Mill 2018; Rand *et al*. 2009; Sefton *et al*. 2007), and others have even found that receiving punishment can reduce a target's propensity to cooperate in the next round (e.g. Anderson and Stafford 2003; Aquino *et al.* 2015; Barclay and Raihani 2016; Bone *et al*. 2015, 2016; Fehr and Rockenbach 2003; Janssen *et al.* 2010; Zheng and Nie 2013). While the proposed reasons given differ according to the experimental setting, one general finding seems to be that the use of punishment is perceived as morally dubious. Therefore, when one player pays a cost to harm another or announces that she will do this if the partner does not bestow benefits upon her, this can erase any cooperative sentiment that might otherwise exist among interaction partners. In other words, the threat of punishment can be said to ‘crowd out’ the motivation to cooperate.

Relatedly, it is unclear to what extent punishment increases cooperation by converting defectors into co-operators (i.e. increasing the number of co-operators), or by increasing the contributions of conditional co-operators (see Kirchkamp and Mill 2018; Lergetporer *et al*. 2014; Shinada and Yamagishi 2007; Yamagishi 1986). In a recent experimental study, the number of free-riders increased under the threat of punishment, relative to a condition where no punishment was possible (Kirchkamp and Mill 2018). By way of explanation for this counter-intuitive result, the authors posit either a possible crowding out effect (where any intrinsic motivation to cooperate might be extinguished by the extrinsic threat of punishment) or the possibility that people expected to be punished and thus attempted to minimise payoff losses ex ante by defecting. Despite the increased numbers of free-riders, total contributions under the threat of punishment were still higher because conditional cooperators made higher contributions under the threat of punishment compared with when no punishment was possible. These results suggest that the threat of punishment might be working more as an assurance to people who are already willing to cooperate as long as they assume others will as well (even if that assumption was mistaken in this specific study), rather than a stick to change the behaviour of free-riders.

The idea that punishment should convert cheaters into cooperators is largely based on classical payoff-based thinking. In economics, it is assumed that individuals try to maximise expected utility and will respond to incentives that change this utility function (like punishment) by changing their behaviour (e.g. by not committing a crime) (Becker 1968). Similarly, behavioural ecologists assumed that natural selection will cause the evolution of strategies that make individuals behave more cooperatively in response to being punished (Clutton-Brock and Parker 1995). Assuming payoff-based decision rules, more severe punishments should act as a stronger deterrent against future offending. Both real-world and empirical studies challenge this prediction. In experimental settings that have manipulated power asymmetries between players, defectors were not more likely to change their behaviour when they were punished by a strong vs a weak partner (Bone *et al*. 2015, 2016). In the real world, it is now well known that harsh sentences, like the death penalty, are no more effective in deterring crime than less harsh ones (Tonry 2018), and a recent quasi-experimental study based on more than 100,000 individuals convicted of a violent crime found that harsher punishments (in the form of prison sentences) were not more effective than lighter punishments (probation sentences) at preventing convicts from re-offending (Harding *et al*. 2019).

Laboratory studies that do report a positive effect of punishment on the future cooperative behaviour of targets also highlight its context-specificity. For example, defectors sometimes respond to light but not harsh punishment (Masclet *et al.* 2003; see also Houser *et al.* 2008; Jiang *et al.* 2013); punishment induces defectors to cooperate in early but not later rounds (Denant-Boemont *et al.* 2007); and the way in which feedback is provided affects how defectors respond to being punished – in a public goods game setting, people are less likely to cooperate in response to being punished if they are shown information about their co-players’ earnings, rather than their contributions (Nikiforakis 2008b). The efficacy of punishment also seems to vary cross-culturally: a meta-analysis of punishment across 18 societies found that the possibility of being punished increased cooperation more in wealthier and high-trust societies than in poorer, lower-trust societies (Balliet and Van Lange 2013). We also note that the same meta-analysis uncovered evidence suggestive of a publication bias in favour of studies finding a positive effect of punishment on cooperation.

There are also apparent methodological issues that may affect the effects of punishment. Most studies that report an overall positive correlative effect of the option to punish on levels of cooperation preclude targets of punishment from retaliating (either because they shuffle group membership each round (e.g. Fehr and Gächter 2000, 2002a) or because the punisher's identity is not revealed to targets (e.g. Fehr and Gächter 2000). If this restriction is removed, punishment frequently prompts retaliation rather than cooperation, particularly when punishers are not anonymous, or if targets can infer who might have punished them previously (e.g. Balafoutas *et al.* 2014b; Bone *et al*. 2015, 2016; Denant-Boemont *et al.* 2007; Dreber, *et al.* 2008; Engelmann and Nikiforakis 2015; Fehl *et al.* 2012; Nikiforakis 2008a; Nikiforakis and Engelmann 2011; Wu *et al.* 2009; Zheng and Nie 2013). Perhaps for this reason, punishment is often inefficient, leading to lower collective payoffs than when no punishment is possible (e.g. Ambrus and Greiner 2012; Aquino *et al.* 2015; Bochet *et al.* 2006; Burnham 2014; Chaudhuri 2011; Egas and Riedl 2008; Gächter and Herrmann 2011; Gächter *et al*. 2017; Hopfensitz and Reuben 2009; Ostrom *et al.* 1992; Rand *et al*. 2009; Vukov *et al.* 2013; Walker and Halloran 2004; Wu *et al*. 2009; but see Gächter *et al*. 2008). The empirical literature on the effects of retaliation is matched by the theoretical literature. For instance, most models exploring the evolution of costly punishment need to prevent retaliatory or antisocial punishment strategies in order to make it work (Rand *et al.* 2010); when these are allowed then punishment typically fails to support the evolution of cooperation (Hauser *et al*. 2014; Janssen and Bushman 2008; Rand and Nowak 2011). Even when retaliation is not an issue, theoretical work also questions the extent to which increased group payoffs can be achieved through punishment owing to the value-destroying nature of punishment (Egas and Riedl 2008; Ohtsuki *et al.* 2009; Vukov *et al*. 2013).

### Prediction 3. Punishers should use punishment in scenarios where it is most likely to deter cheating

Punishment may have a deterrent effect if it reduces the probability that a cheat (or an observer) will re-offend in future. There is a subtle but important distinction between special and general deterrence, the former being the aim to deter the offender from re-offending and the latter being the aim to deter any would-be offender from committing the same crime (Bentham 1962; Hoffman 2014). Punishment could in principle be consistent with either of these deterrent effects. People often report that the justification for punishment decisions is the beneficial deterrent effect (Carlsmith *et al.* 2002; Cushman 2015; Mathew and Boyd 2014), and some empirical work supports the idea that punishment is indeed administered with (special) deterrence in mind. For example, people are more likely to invest in third-party punishment when they believe that the cheat would also harm them (Krasnow *et al.* 2016). If mistreatment of the victim does not predict mistreatment of oneself, then people are less likely to invest in third-party punishment.

Punishment as a special deterrent predicts that people should be more likely to punish: (i) intentional harm; (ii) in scenarios where a behaviour change effect is theoretically possible; and (iii) where recidivism is likely. Individuals with nefarious intent should be more likely to be punished because these bad intentions are a reliable indicator that the target is likely to re-offend (although there can be scenarios where harm was not intended but punishment might still deter future offending, for instance, a driver of a speeding car might not intend to hit a pedestrian but can nevertheless be punished and potentially deterred from speeding in future). Under the deterrence hypothesis, punishment should be preferentially aimed at individuals who can change their behaviour because there is little to be gained from punishing someone who cannot change. Finally, the deterrence hypothesis predicts that the potential for recidivism should be important for similar reasons: there is little to be gained from punishing someone who is unlikely to re-offend in future. In contrast to these predictions, however, empirical data suggest that, although people are sensitive to intentions when making punishment decisions, they also punish bad outcomes produced from benign intentions (Cushman *et al*. 2009; Martin and Cushman 2016) or from random processes (Houser and Xiao 2010). In addition, people often punish in scenarios where future cooperation from the target is impossible, for example in one-shot settings (Bone and Raihani 2015; Fehr and Fischbacher 2004; Raihani and McAuliffe 2012; Tan and Xiao 2018; Walker and Halloran 2004), in games where group membership is shuffled between rounds such that punishers do not meet targets again (Choi and Ahn 2013; Fehr and Gächter 2002a), when people know it is the last round of a social interaction (Choi and Ahn 2013; Decker *et al*. 2003; Engelmann and Nikiforakis 2015; Faillo *et al.* 2013; Gächter *et al.* 2008; Ones and Putterman 2007) or when punishers themselves terminate the interaction (Barclay and Raihani 2016). People also punish even when the punishment will not be communicated to the target and cannot therefore deter future cheating (Crockett *et al*. 2014; Fudenberg and Pathak 2010). Punishment decisions are often unrelated to the potential for recidivism: in a study using vignettes to gauge whether putative transgressors ought to be punished, participants paid little attention to the likelihood that the target would re-offend even if punishment was not administered (Darley *et al.* 2000). Finally, punishment as a special deterrent should be sensitive to mitigating circumstances: in contrast, ethnographic accounts suggest that even when people have strong extenuating circumstances for defection, they may still be punished for doing so (Mathew and Boyd 2014).

While much observed punishment therefore seems inconsistent with the strategic aim of changing the behaviour of targets, it is nevertheless possible that punishment serves as a general deterrent: to deter any potential partner from cheating. Punishment as a general deterrent should be less sensitive to the intentions or culpability of the target, so long as observers believe that an intentional crime was committed (Carlsmith *et al.* 2002). A model of general deterrence predicts that punishment is only administered when it is visible to others. Some work has found that third-party punishers are sensitive to the presence of an audience, being more likely to punish when observed (Kurzban *et al.* 2007). However, in this setup, the interaction was one-shot and so there was no deterrent benefit to be gained from advertising investments in punishment. In a more recent two-player helping game, where recipients could punish unhelpful donors, people tended to invest more in punishment when their punitive reputation would be advertised to future partners. Moreover, donors were more likely to cooperate when playing with a partner with a punitive reputation (dos Santos *et al.* 2013). These findings support the idea that punishment can have a general deterrent effect and is administered with this effect in mind. To our knowledge, there has been no experimental work exploring whether punishment in multiplayer games is also administered with general deterrence in mind. For instance, in a public goods game, people may punish not just to harm cheats but also to show other group members that cheaters will be punished. To test this, we would need to explore whether investments in punishment in public goods games are sensitive to whether the punishment is communicated to other group members. It may be important to separate the information about the punishment and the punisher's identity: people may want others to know that punishment has occurred but not necessarily to be identified as the punisher because this might make the punisher a target for retaliation or potential eviction from the group (see Rockenbach and Milinski (2011), who found that people paid to hide the fact that they punished other group members in a setting where players could be evicted from the group). Some real-world data on punishment in multiplayer settings has been interpreted in terms of general deterrence: in the semi-nomadic Turkana tribe of northern Kenya, age-mates physically punish a peer who does not participate in or shows cowardice during a battle with the neighbouring ethnic group (Mathew and Boyd 2011). Such punishment has been interpreted as providing an incentive to men who might otherwise defect to participate in these dangerous battles (i.e. a general deterrent effect), but to show this conclusively would require a comparison with a case where no punishment was possible. Moreover, it is possible that there are other personal benefits to be gained from participating in raids that might incentivise men to contribute, including the possibility to directly increase their own resources, to be chosen as a social partner and to improve their success in the mating market (Baumard and Liénard 2011). That men stood to directly benefit in their participation in raids was also supported in follow-on work using vignettes to explore people's opinions of free-riders (Mathew and Boyd 2014).

Despite some examples of punishment being consistent with a deterrent aim, some problematic findings remain. For example, the findings listed above – indicating that people punish at the end of interactions or when they terminate the interaction – are also inconsistent with a general deterrent aim, at least if deterrence should operate within the confines of the experimental setting. One could argue that people do not bring a psychology for one-shot, anonymous interactions into these experiments (the ‘big mistake’ or mismatch hypothesis; Cosmides and Tooby 2006) – and that punishment could therefore still be consistent with a general deterrent aim. Nevertheless, several studies find that punishment is higher in the last round of an interaction than in preceding rounds (Faillo *et al*. 2013; Gächter *et al.* 2008; Guala 2012; Page *et al.* 2013). While the motivation behind such adjustment is unknown, the results show that people do adjust their behaviour to the possibility of future interactions, although in opposite ways to predictions based on a deterrent function of punishment.

### Prediction 4. Punishers should target defectors, not cooperators or those that did nothing wrong

If punishment functions as a tool to promote cooperation, it should be (mostly) aimed at defectors and seldom at cooperative individuals. While most empirical evidence suggests that this is the case, in many experimental settings there are non-negligible levels of punishment directed towards people who did nothing wrong (e.g. Abbink and Herrmann 2011; Abbink and Sadrieh 2009; Dawes *et al.* 2007; Paál and Bereczkei 2015; Raihani and McAuliffe 2012; Wu *et al.* 2009) or towards those who cooperated more than the punisher (Anderson and Putterman 2006; Cinyabuguma *et al.* 2006; de Melo and Piaggi 2015; Falk *et al.* 2005; Goette *et al.* 2012; Herrmann *et al.* 2008; Irwin and Horne 2013; Pfattheicher *et al.* 2017; Pleasant and Barclay 2018; Sylwester *et al.,* 2013). In some societies, cooperators are almost or equally as likely to be punished as defectors (Gächter *et al.* 2010; Gächter and Herrmann 2011; Herrmann *et al.* 2008). The tendency to punish cooperators has been found to vary across societies (e.g. Herrmann *et al.* 2008) and among individuals. For instance, Falk *et al.* (2005) found that, in a prisoner's dilemma game, cooperative individuals tended to target punishment at defectors, whereas defectors targeted cooperators and defectors more or less equally. Moreover, in this study, cooperators continued to use punishment even when it could not change the payoffs between themselves and a partner, whereas defectors did not use punishment under these circumstances.

### Prediction 5. Punishers should respond to harmful actions not to payoffs per se

Punishers who have the strategic aim of deterring cheats should be primarily sensitive to experiencing losses, whereas punishers who have the strategic aim of restoring equality or competing with the partner should be more sensitive to payoff differences between themselves and the target (a psychological predisposition that has also been called inequity aversion, Fehr and Schmidt 1999). In most social dilemmas, interacting with a cheat means that partners incur losses and experience disadvantageous inequity (Raihani and McAuliffe 2012), meaning that these motives cannot easily be disentangled. Studies which have attempted to isolate the proximate basis of punishment decisions in the laboratory found that punishment decisions are at least (or sometimes more) sensitive to inequity than to losses and suggest that punishment might often be motivated by disliking being worse off than others, rather than disliking being cheated (Bone and Raihani 2015; Carlsmith *et al.* 2002; Dawes *et al.* 2007; Gächter *et al.* 2017; Houser and Xiao 2010; Johnson *et al.* 2009; Masclet and Villeval 2008; Paál and Bereczkei 2015; Raihani and McAuliffe 2012).

For example, Raihani and McAuliffe (2012) devised an experiment involving a two-player game where one player could steal from the other. Importantly, in this game, the victim of theft always incurred the same loss but the relative outcomes varied across three conditions where, after stealing, the thief remained at a relative disadvantage, had the same payoffs as the victim or became better off than the victim. This study showed that the primary factor determining whether the victim would punish the thief was whether the thief ended up better off: incurring losses in the absence of relative disadvantage did not motivate individuals to punish. Similar findings have been obtained with capuchin monkeys (Leimgruber *et al.* 2016). In the context of third-party punishment, one recent study found that punishment decisions were better predicted by the envy experienced by the punisher than by any moralistic outrage felt on behalf of the victim (Pedersen *et al*. 2013; see also Leibbrandt and Lopez-Pérez 2012; Paál and Bereczkei 2015). In another recent study that took a slightly different approach, Sznycer *et al*. (2017) ran a series of questionnaires about wealth re-distribution to large-*N* samples of participants from the US, the UK and India. Around 14–18% of participants stated that they would prefer a tax policy that removed 50% of earnings from the wealthiest individuals in society over and above a policy that removed 10% of earnings, but nevertheless produced more money for the poor (because the wealthier people earned more under this policy). Finally, other work shows that punishment is used by punishers to create a payoff advantage relative to targets. For example, in Bone and Raihani (2015), people preferred to use punishment to create equal outcomes when possible but when equal outcomes were not possible, punishers frequently chose the harshest punishment possible, creating the largest asymmetry between their own and target's payoffs. This is strongly suggestive of a competitive strategic aim (a pattern also reported in Houser and Xiao 2010).

Many of the patterns described above (e.g. punishing in the last round of an interaction, punishing when it cannot be communicated to the target) are more consistent with a competitive strategic aim than with a deterrent aim. Competitive punishment strategies should only be used when punishers can change the payoff differences between themselves and a target: in accordance with this prediction, previous studies have reported that antisocial punishment is extremely rare (relative to ‘justified’ punishment) when fee-to-fine ratios of 1:1 are used (Bone *et al.* 2015, 2016; Bone and Raihani 2015; Egas and Riedl 2008; Falk *et al.* 2005; Sylwester *et al.* 2013). Previous work has also indicated that the tendency for antisocial or other competitive punishment strategies is exacerbated under resource scarcity (Prediger *et al.* 2014), and is higher in societies with lower GDP and weaker norms of civic cooperation (Herrmann *et al.* 2008), suggesting that investments in competitive punishment might ultimately relate to ecology and the scale of competition. Competitive punishment strategies might be most likely to evolve under resource scarcity because the benefits of harming or eliminating a competitor are greater when resources are scarce (e.g. see Sznycer *et al.* 2017), and in environments where competition is relatively local rather than global (see for supporting evidence Barclay and Stoller 2014; Barker and Barclay 2016).

## 3. Brief summary of findings

The findings above indicate that: (i) punishers are not always cooperative; (ii) being punished does not consistently have the expected effect of increasing cooperation from the target, or of increasing group payoffs; (iii) cooperators as well as defectors can be targeted for punishment; and (iv) punishers are often proximately motivated by payoff differences rather than by cheating or losses per se. These findings are problematic from a perspective that views punishment solely as a tool to promote cooperation but are consistent with the hypothesis that punishment serves a competitive function. The fact that punishment can be associated with different strategic goals also helps to illuminate how punishers are perceived. The aims of general deterrence and a desire to create equal outcomes among all players (egalitarianism) are both, broadly speaking, aims that are consistent with producing public goods. Thus, if people infer that punishment has these collective goals in mind, then they should generally approve of punishers. In contrast to this prediction, most published studies indicate that punishers are generally disliked and disapproved of (reviewed by Raihani and Bshary 2015c), and that punishers might only be approved of in a very restricted range of circumstances where competitive or self-serving motives can be effectively ruled out (see Barclay 2006; Jordan *et al.* 2016; Kiyonari and Barclay 2008; Raihani and Bshary 2015b, c). We suggest that punishers may often be disliked or disapproved of because punishment is also often consistent with a competitive strategic aim, whereby the punisher seeks to increase their own payoffs relative to those of the target.

The possibility that punishment can result in immediate fitness benefits to punishers – without the need for any conditional response from targets – somewhat blurs the distinction between punishment and what we (and others) had previously labelled ‘sanctions’ (interactions where aggressors derive immediate benefits from their actions, also known as negative pseudo-reciprocity; Bergmuller *et al.* 2007; Raihani *et al*. 2012a) or negative indirect reciprocity Bhui *et al.* 2019). Under negative pseudo-reciprocity (sanctions), investments in harming another individual can be self-serving even if the target does not change his behaviour. For example, female coral gobies have a linear size-based dominance hierarchy and may evict any subordinate who grows too close in size to them (Wong *et al.* 2007). Previously we argued that this did not fit the definition of punishment, as the evictor gains a direct benefit when she removes a competitor that does not rely on the competitor behaving more cooperatively in future interactions with the evictor (Raihani *et al*. 2012a). However, it is now apparent that many examples of punishing in humans also have the same property: despite involving a short-term payoff-reduction, punitive strategies can be fitness-enhancing if they change the relative payoffs of punishers and their targets. Many ethnographic accounts describe how deviants or cheats risk being ostracised or deserted by fellow group-members, and there are also many examples of homicidal or otherwise dangerous individuals being executed by other members of the group (Boehm 2011; Wrangham 2019). These kinds of harmful acts that also terminate the interaction between cheats and victims would involve similar payoff structures as eviction in the goby example above. To account for the variety of potential direct and indirect fitness benefits, we propose that the definition of punishment should focus on the act itself: an act that lowers the immediate payoff of actor and recipient, rather than the circumstances that trigger punishment or the consequences in terms of the target's behaviour.

## 4. Punishment may be rare, but punitive preferences are not

A competitive account of punishment can potentially help to reconcile the fact that punishment of the variety we study in laboratory settings seems to be relatively rare in the real world, while accounting for the expression of punitive sentiment that is more frequently observed. For instance, ethnographies suggest that a common expression of punitive sentiment occurs in the form of mild criticism or mocking the target (Baumard 2010; Boehm 2011; Wiessner 2005). For example, Hoebel (1954, p. 93) described how conflicts among Inuit could be resolved with song-duels, where accused and accuser took it in turns to ridicule one another in verse. Similarly, Ju/’Hoansi bushmen mediate conflict and express displeasure with others by using various forms of reputational put-downs, including mocking, ridicule and outright criticism, rather than physical aggression (Wiessner 2005). A parallel example comes from studies of the Cheyenne, where shaming of thieves occurs by a statement to the effect of ‘If I had known you wanted that thing, I would have given it to you’ (Hoebel 1954, p. 169). Notably, it is not only defectors or cheats who are targeted for shaming and ridicule but also individuals who have or seek status or prestige. Indeed, Wiessner (2005) writes that a key function of mocking and ridicule is to ‘level big-shot behaviour’. Perhaps because cooperative acts are associated with reputational gains for the cooperative individual, ostensive or showy acts of generosity are often hidden (Raihani 2014), ignored (Wiessner 2005) or even ridiculed (e.g. ‘do-gooder derogation’; Minson and Monin 2012), findings which also speak to the competitive underbelly of punitive sentiment.

Other ways that punishers can inflict reputation or status costs on targets is via gossip (Beersma and Van Kleef 2012; Dunbar 2004). Gossip is prevalent in real-world settings and seems to have similar payoff structure to punishment in that it is costly to be perceived as a gossip (Adams and Mullen 2012) and to be gossiped about (Feinberg *et al*. 2012). Gossip typically refers to conversation or statements that convey social information about absent third parties. A disproportionate amount of human speaking time (~65%, with little variation across cultures, ages or gender; Dunbar 2004) is used to gossip. Gossip can be used to convey or gather information about interaction partners in the absence of direct observation and individuals are sensitive to the potential effects of gossip on their reputation, behaving more cooperatively when reputation is at stake (Feinberg *et al*. 2012, 2014; Jolly and Chang 2018; Wu *et al.* 2016a, b).

While gossip can in theory transmit either positive or negative information about a target, humans apparently prefer to gossip negatively (Feinberg *et al.* 2014; Peters *et al*. 2017). Like punishment, negative gossip is subjectively rewarding to gossipers and is elicited by the same negative emotions (i.e. frustration and anger at interacting with a cheat) that produce punishment (Feinberg *et al.* 2012). As with punishment, it seems that there is also the potential for gossip to stem from different strategic aims. For instance, Feinberg *et al*. (2012) show that tendency to gossip is associated with prosocial value orientation and people state that the reason for gossip is to help warn the recipient about the previous untrustworthy behaviour of a confederate. However, others have shown that the tendency to gossip negatively is predicted by personality traits implicated in antisocial preferences, including psychopathy and Machiavellianism (Lyons and Hughes 2015). In her study of the Ju/’hoansi bushmen, Wiessner (2005, 2009) found that ~30% of cases of gossip were ‘unfounded and concocted out of jealousy or social strategies’ (P. Wiessner, personal communication). Similarly, empirical work has shown that gossip is most likely to occur in triadic settings, with two people gossiping about a higher-status other (Ellwardt *et al.* 2012). As with punishment, it is unlikely that gossip functions to induce cooperation from the target towards the gossiper in the future (i.e. special deterrence), although it might be the case that gossipers are motivated by a more general deterrent aim. One might additionally posit that gossipers, like punishers, are motivated by retributive or competitive aims (i.e. to damage a defector's reputation to increase gossiper's relative status and improve the gossiper's relationships with the audience, e.g. Bosson *et al.* 2006; Jolly and Chang 2018; Peters *et al*. 2017). These hypotheses are amenable to empirical testing.

## 6. Institutionalising punishment

Given the difficulty in correctly pinpointing why someone punishes, and what their strategic aim might be, it is perhaps unsurprising that we do not typically see participants in experimental games responding to punishment by increasing cooperation. We suggest that the way people respond to punishment is likely to hinge fundamentally on the motives and aims they attribute to punishers – and that these attributions will hinge crucially on the features of the interaction – broadly speaking, whether the punisher can potentially derive personal benefits from their actions. As far as we know, the link between features and attributions has seldom been explored in empirical studies and thus represents a fruitful avenue for inquiry (see Ho *et al*. 2019 for a first step in this direction). The tendency to infer that punishers are driven by cooperative or competitive motives is expected to vary with the context of punishment (specifically whether the context allows that the punisher can benefit from their actions, for example, if punishers expect to interact with targets in future, or can improve their own relative payoffs through the act of punishing – this is discussed at length in Raihani and Bshary 2015c). The tendency to attribute competitive motives to punishers might also vary across individuals. For instance, previous research has shown that there is enormous variation in the general population in the tendency to attribute malevolent intentions to others in ambiguous social settings (Raihani and Bell 2017a; Saalfeld *et al.* 2018) and that the tendency to attribute malevolent intentions to others also predicts punitive responses in social interactions (Raihani and Bell 2017b). In the standard laboratory setting, where individuals receive punishment in the form of monetary fines without any explanation for the basis of the punishment nor agreement upon the actions that warrant punishment and how severe punishment should be, there is enormous scope for variation and error when targets attempt to interpret a punisher's intentions. For instance, targets might infer that the punisher dislikes interacting with a cheat and wants them to cooperate in the next round. However, a target of punishment might also reasonably infer that the punisher simply wishes to inflict harm rather than to promote cooperation. The intentions attributed to punishers might also vary with societal norms surrounding the use of punishment. For instance, in societies where individuals expect others to cooperate, punishment might be viewed as a legitimate response to defection, with the inferred intention being that punishers seek to change defectors’ behaviour. In societies where such norms do not exist, punishers might be more likely to be perceived as having competitive aims, with the result that punishment is less likely to foster cooperation (see discussion in Balliet and Van Lange 2013). Empirical work addressing whether intention attribution affects responses to punishment in economic games would be useful to explore if and how intention attribution affects targets’ responses to punishment.

Suggestive evidence that the perceived intentions of punishers do affect targets’ responses comes from empirical studies where punishment can be construed as being driven to a greater or lesser extent by self-interested motives: in a two-player Trust Game, trustees who demanded a large investment from the partner under the threat of punishment received less than those who did not issue a punishment threat (Fehr and Rockenbach 2003; see also Houser *et al.* 2008). Other work indicates that people attempt to infer the proximate motives underpinning punishment decisions and respond accordingly. Xiao (2013) used a sender–receiver game, where the sender could benefit by deceiving the receiver, and introduced a punishment stage where the receiver could additionally punish the sender. In some treatments, receivers immediately benefitted from punishing the partner, whereas in others punishment was associated with a payoff reduction. When receivers could benefit from punishing the sender, third-party bystanders were less likely to infer that the sender had cheated, compared with cases where receivers could not benefit from punishing the sender (Xiao 2013). Similarly, a meta-analysis finds that decisions to reduce someone else's payoffs are more likely to promote cooperation when these payoff reductions are costly to the actor, rather than free, to implement (Balliet *et al.* 2011).

Generally, we predict that the tendency to cooperate in response to punishment will be inversely related to the extent to which targets of punishment can infer that the punisher has competitive aims. Cooperating or acquiescing in response to competitive punishment can be construed as capitulating in response to aggression from an evenly matched peer (as punishment is typically operationalised in experimental economic games). Instead, under natural conditions, there might be selection against targets allowing themselves to be subordinated, and individuals might attempt to preserve their status by retaliating against evenly matched aggressors (see Boehm 2011). Indeed, the rejection of unfair Ultimatum Game offers has been interpreted as a means of avoiding acquiring a subordinate status (Yamagishi *et al.* 2012) and punishment is also linked to testosterone levels in men (Burnham 2007), as would be expected if punishment is viewed as a dominance competition rather than as a way to communicate cooperative norms. Selection against subordination could be stronger if capitulating to aggressors is also observed by others. In much the same way as gaining a punitive reputation can yield an advantage to punishers (Barclay 2006; dos Santos *et al*. 2011, 2013; dos Santos and Wedekind 2015; Raihani and Bshary 2015b, c), a reputation for being easily subordinated might be disadvantageous to acquire (see Cohen *et al.* 1996; Crombag *et al*. 2003; Osgood 2017). This hypothesis deserves further theoretical and empirical attention.

The possibility for punishment to spark retaliation, rather than cooperation, is prevalent in historical (Barrett *et al.* 2005) and ethnographic records (Boehm 2011; Hoebel 1954; McCullough *et al.* 2013; Wiessner 2005; see Jackson *et al.* 2019 for a recent review). Indeed, many societies seem to have independently converged on customs (e.g. duels and other ritualised contests) that circumscribe the contexts in which punishment can be administered, perhaps to limit these detrimental consequences (Boehm 2011). As a consequence, decentralised peer punishment of the variety studied in experimental economic games is often rare or absent among humans in the real world (Guala 2012; Pedersen *et al.* 2018; Wiessner 2005), although it tends to be more common in contexts where such customs are lacking (reviewed in Jackson *et al*. 2019). These findings speak to a more specific insight, which is that for punishment to be effective as a tool to convert cheaters into co-operators, it must be perceived as legitimate (Baldassarri and Grossman 2011; Bowles and Gintis 2013; Ertan *et al*. 2009; Faillo *et al.* 2013; Gross *et al.* 2016; Tyler 2006; Villatoro *et al.* 2014; Xiao and Tan 2014; Zheng and Nie 2013). Here, we define legitimisation as any process, mechanism or institution that reduces the scope for punishment to be driven by competitive aims and thereby increases the probability that targets cooperate in response to punishment.

Perhaps the simplest legitimising mechanism is communication: allowing people to explain to targets why they are punishing means that cooperation is more likely to ensue (Janssen *et al.* 2010). Legitimacy can also be promoted by more formalised agreements or customs, which are often collectively referred to as institutions (Cushman 2015; Hurwicz 1996; North 1990; Powers *et al.* 2016). Specifically, institutions can be thought of as political game forms which change the payoffs associated with social interactions (Hurwicz 1996; Powers *et al.* 2016). Institutions probably play a vital role in changing the game form of punishment from one where individuals can benefit by harming others, regardless of how the target behaves (i.e. as is the case with competitive punishment) to one where it only pays to punish cooperative norm violators (Boyd 2017). In much the same way, it has been posited that institutions played (and continue to play) a vital role in transforming social dilemmas (where the strategic incentive is to defect) into games where the individually self-interested strategy is to cooperate (Boyd 2017; Powers *et al.* 2016; Sigmund *et al.* 2010). Although institutions might thus generate benefits for all members of a group, we also note that the historical record contains many examples of institutions being used to further the interests of a powerful few at the expense of the many (Acemoglu 2006; Acemoglu *et al.* 2001; Briggs *et al.* 2005).

Crucially, institutions have often been omitted from standard experimental economic investigations of peer punishment, which might help to explain the relatively mixed results on the efficacy of peer punishment in these stylised settings. One way to legitimise punitive acts is for participants to self-select into groups with punishment regimes (see Gürerk *et al.* 2006 for experimental evidence and Frey and Sumner 2019 for a real-world example), to vote for sanctioning institutions (Sutter *et al.* 2010) or to be involved in shaping institutions that dictate how punishment will be implemented (Decker *et al.* 2003; Ertan e*t al*. 2009). Recent experimental work indicates that the requirement for consensus is an important institution for legitimising punishment: when punishment decisions are reached by consensus, punishment is more effective at promoting cooperation (Cardenas 2000; Casari and Luini 2009; Eriksson *et al.* 2017; Ertan *et al*. 2009; Hilbe *et al.* 2014; Pfattheicher *et al.* 2018; Shinada and Yamagishi 2007; Villatoro *et al.* 2014; Zheng and Nie 2013). Many laboratory studies reporting positive effects of punishment on targets’ subsequent behaviour (e.g. Fehr and Gächter 2000, 2002a; Pfattheicher *et al*. 2018; Rockenbach and Milinski 2006; Shinada and Yamagishi 2007) do not report how many people independently punished a cheating player, meaning that it is unclear to what extent any positive effects of punishment on the target's subsequent cooperation were driven by these seemingly consensus-based cases in which two or three peers decided independently to punish the same target.

Data from real-world settings also support the idea that consensus-based legitimacy can increase the efficacy of punishment. For example, Mathew and Boyd (2011) argue that punishment is only administered after a consensus-making process, noting that ‘vigilante’ punishment that is administered without prior consensus is disapproved of. Similar historical examples also exist. In medieval England, men were organised into small groups called ‘tithings’ who were responsible for administering punishment to any defector in their group. Interestingly, these real-world examples show that participation in group punishment is often (i) forced rather than voluntary and (ii) administered by the allies or kin of the target (Hoebel 1954, p. 89). Both features might mean that there is less scope for targets to infer that punishers are motivated by competitive aims and/or benefit via enhancing their own relative payoffs in such interactions (see also Boehm 2011 for ethnographic examples). Moreover, consensual punishment administered by group might reduce the scope for retaliation (and feuds) simply by virtue of the cost associated with retaliating against a larger group. Legitimacy is also helped by restricting who can punish: when people can only punish those who contribute less than them (Faillo *et al*. 2013; Grieco *et al.* 2017), it reduces the possibility that punishment is perceived as a competitive action.

Outsourcing punishment to authorities (also called ‘centralising’ punishment) might be another way to increase legitimacy. When given the choice, people prefer to pay taxes to provide centralised ‘pool’ punishment rather than peer punishment (Andreoni and Gee 2012; Traulsen *et al*. 2012), although other work finds that, when given the choice between a punishment or no punishment regime, many people at least initially opt for the latter (Guillen *et al.* 2007; Gürerk *et al.* 2006). Moreover, when a centralised punishment mechanism is available, people reduce their investments in peer punishment, leading to overall higher average payoffs (mainly because people are not paying for peer punishment and retaliation is reduced in the centralised punishment regime; Andreoni and Gee 2012). Nevertheless, evidence that centralised punishment is more effective than peer (decentralised) punishment is mixed (Andreoni and Gee 2012; Baldassarri and Grossman 2011; Carpenter *et al.* 2012; Grieco *et al.* 2017; Nosenzo and Sefton 2014; O'Gorman *et al.* 2009; Traulsen *et al.* 2012), with a meta-analysis indicating that centralised punishment is actually less effective than decentralised punishment at promoting cooperation (Balliet *et al*. 2011). We predict that the perceived legitimacy of the authority will be crucial for determining whether the punishment they deliver is effective, and thus increases group welfare. For example, an experiment in Uganda found that participants were more than twice as likely to increase cooperation in response to punishment from an elected monitor than in response to punishment from a monitor who was randomly assigned to the role (Baldassarri and Grossman 2011). Often, individuals with the power to wield punishment are either elected to that role on the basis of their character and can be demoted if they are perceived to be corrupt (Lierl 2018) or are held in check by a variety of mechanisms (institutions) that preclude or strongly disincentivise corrupt leadership methods (Bøggild and Petersen 2016).

We also predict that centralised punishment will be more effective where there is little potential for authorities to obtain personal benefits from their punitive acts. Sometimes, authorities can benefit from the punishments that are administered – for example, Hoebel (1954) describes how Inuit shamans acting as authorities in disputes involving women would often instruct the woman to have intercourse with him (his powers being conveniently able to counteract the effects of her sinning). Similarly, in many small-scale societies, monitoring is incentivised by allowing individuals who detect crimes to keep a portion of any fine that is levied on a cheat (Ostrom 2015). In the Ifugao of Luzon, people known as monkalum act as informal go-betweens in disputes and conflicts, and benefit in this quasi-judiciary role from taking fees from defendants (Hoebel 1954, p. 116). In such situations, we expect that even punishment from authorities will be scarcely better than conventional peer punishment in promoting cooperation. Punishment is more likely to have a cooperation-enforcing effect when authorities are perceived to be impartial (Muthukrishna *et al.* 2017). Indeed, a recent experiment conducted in Liberia found that punishment from leaders only promoted cooperation when leaders received a flat fee for the role of punisher, rather than benefitting from administering the punishment itself (Beekman *et al.* 2018). Similarly, experimental evidence from real-world leaders of a forest commons management programme in Ethiopia revealed that: (i) leaders varied in their propensity to punish indiscriminately (i.e. competitively) in an experimental setting; and (ii) competitive leaders were less effective at promoting collective cooperation in the context of the forest management (Kosfeld and Rustagi 2015). Procedural fairness thus seems to be an important device for increasing legitimacy (Bøggild and Petersen 2016; Cremer and van Knippenberg 2003; Eisner *et al.* 2017).

## 7. Moving forwards

We believe that new theoretical and empirical studies are now needed to examine the conditions under which punishment with different strategic aims outlined above (Table 1) could be favoured by selection. In this endeavour, we suggest that identifying the relevant ecological and social factors that affect the use and efficacy of punishment will be crucial. These factors are likely to include (but are not limited to) population structure, resource availability, the likelihood of repeated interactions, the scale of competition and the strength and content of the institutions surrounding the acceptable use of punishment. We predict that competitive punishment should be increasingly favoured by selection as the scale of competition becomes increasingly local, although we also believe that more work is currently needed on how ecology (resource availability, population structure, pathogen prevalence, environmental predictability, etc.) affects the scale of competition and the form and content of institutions across societies. Ideally, theoretical models would also start to explore the evolutionarily stable responses to punishers (as in Hilbe and Traulsen 2012; Morris *et al.* 2017). For example, it would be helpful to know under what conditions cooperating in response to punishment might be under negative selection (readily submitting to peers might be under negative selection when observed by others, for example).

**Table 1.** Strategic aims underpinning punishment and the problematic findings from the published literature that are inconsistent with each goal

Strategic aim	Problematic examples
Special deterrence	People often punish unintentional harm. People punish even when no special deterrence is possible e.g. one-shot interactions/end of interaction. People punish when target will not know they have been punished. People punish those who have done nothing wrong/cooperated. People often punish without concern for likelihood of recidivism. People punish even when there are strong mitigating circumstances. Tendencies to punish cheats and to reward co-operators are often uncorrelated.
General deterrence	People punish in the last round of interactions, where no general deterrence benefit is possible. Punishment is often higher in the last round than preceding rounds. People often do not approve of punishers. People punish in the absence of reputational effects. People punish even if that is not communicated to third parties.
Egalitarian	People often use punishment to create inequality. Punitive and cooperative tendencies are often unrelated.
Competitive (enhance own payoffs)	People do still use punishment, even when 1:1 fee to fine ratio, or when punishment is more costly to the punisher than to the target. People prefer to compensate victims, if given the choice. People sometimes use punishment to create equal outcomes when this is possible.

In empirical studies, we believe it is important that punishment is implemented using various cost ratios (e.g. as in Bone and Raihani 2015; Bone *et al.* 2015, 2016; Egas and Riedl 2008; Nikiforakis and Normann 2008; Sefton *et al.* 2007). We are not aware of any biological rationale for the commonly implemented 1:3 fee-to-fine ratio. In fact, giving each person in a dilemma this same technology makes little biological sense. A 1:3 ratio implies a dominance hierarchy or a power asymmetry (it costs me a small amount to inflict a larger cost on you): it is not obvious how an individual can be both a dominant and a subordinate in such a power struggle. More generally, we also note that the standard 1:3 fee-to-fine ratio has the competitive function inbuilt by default. While some authors have argued that low-cost, high-impact punishment is necessary to promote cooperation (Chaudhuri 2011; Nikiforakis and Normann 2008; but see Balliet *et al.* 2011), it is also possible that these competitive fee-to-fine ratios are more likely to prompt retaliation, thereby undermining cooperation. We suggest that work exploring punishment that is more costly to administer than it is to receive might be more likely to be interpreted as an honest signal of discontent (rather than as an expression of competitive motives) and might thus be more likely to induce targets to cooperate (see Balliet *et al.* 2011 for evidence on the importance of punishment costs for promoting cooperation).

An underexplored line of research is to compare punishment in humans explicitly with that in other species. Few studies have adapted typical human laboratory games to test for punishment decisions in primates (Jensen *et al.* 2007a, b; Leimgruber *et al.* 2016), and even rarer are studies that explore to what extent punishment may stabilise human cooperation under conditions in which punishment stabilises cooperation in other species (but see Bone *et al.* 2016). One important theme in the cleaner fish mutualism seems to be asymmetries. Among cleaners and their clients, there are clear asymmetries in strategic options owing to pre-determined roles: the cleaners are service providers that can cheat while the clients are what the name implies, lacking options to cheat in return (unless they are predators and hence could eat the cleaner; Trivers 1971). In the case of male cleaners punishing cheating female partners, there are asymmetries in power among interacting players as male cleaners are larger than and dominant to female partners. Indeed, females never punish males for cheating a shared client. In the vervet monkey example, punishment seems to be used as a form of negotiation over what actions to take, as females have more to gain than males from escalating and attacking the neighbouring group. Thus, it appears that intergroup encounters are not about cooperating and free-riding but about diverging interests between the sexes. Capturing key features of these animal examples in new laboratory-based experiments may yield important insights about punishment in humans, no matter whether results will be similar or different from the animal cases (Bone *et al.* 2016).

We also need more studies which tease out possible motives underpinning punishment decisions and responses to punishment. For example, if punishment serves a competitive function, then helpful individuals who reap disproportionate payoffs from their actions should be more likely to be punished than unhelpful individuals who nevertheless remain at a relative disadvantage – and individuals might therefore avoid any actions (not just cheating) that leave them at a payoff advantage over partners in social interactions.

To conclude, we suggest that human punishment might sometimes or by some persons be aimed at changing the behaviour of targets but that this is not always the case – and that competitive motives are understudied and yet arguably more important. There is more evidence to suggest that the possibility of punishment acts as an antecedent deterrent against would-be cheats than as a tool to convert cheats into cooperators – but this could be an exaptation, with the primary function of punishment being to reduce personal payoff disadvantages or improve punisher's own payoffs relative to the target's. Certainly, the existing empirical evidence on the proximate motives underpinning punishment decisions suggests that punishment decisions frequently stem from fairness and/or status concerns, rather than from the desire to reciprocate harm. Researchers interested in the role of punishment in supporting human cooperation – including us! – need to start thinking hard about additional possible functions of punishment in humans.
