# Punishment: one tool, many uses – CORRIGENDUM

**DOI:** 10.1017/ehs.2019.18

**Published:** 2020-02-10

**Authors:** Nichola Raihani, Redouan Bshary

**Keywords:** Competition, cooperation, fairness, punishment, spite, corrigendum

The authors apologise for missing the inclusion of the citation for a passage on page 4-5 for *Hare, D., Reeve, H.K. and Blossey, B.* (2016) with their article.

Additionally, a recent theoretical model on the evolution of cooperation (which was modelled as respect for resource possession) found that cooperation was stabilised when resource holders (or other individuals) responded to ‘raiding’ (attempts to steal the resource) with retaliation (i.e. punishment). Nevertheless, when the level of inequality between resource-holders and resource-seekers exceeds a critical threshold (which can be thought of as akin to variation in payoff differences among players in a laboratory game), then cooperation collapses and raiding strategies come to dominate the population until this inequality is reduced. This model, although not explicitly framed in terms of cooperation and punishment, illustrates the evolutionary logic of considering relative payoff differences in understanding the evolution of cooperation and punishment in nature. (Hare *et al.* 2016).
